# Neuregulins in Neurodegenerative Diseases

**DOI:** 10.3389/fnagi.2021.662474

**Published:** 2021-04-09

**Authors:** Guan-yong Ou, Wen-wen Lin, Wei-jiang Zhao

**Affiliations:** ^1^Center for Neuroscience, Shantou University Medical College, Shantou, China; ^2^Cell Biology Department, Wuxi School of Medicine, Jiangnan University, Wuxi, China

**Keywords:** neuregulin, ErbB receptor, neurodegeneration, neurodegenerative diseases, treatment

## Abstract

Neurodegenerative diseases, including Alzheimer’s disease (AD), Parkinson’s disease (PD) and amyotrophic lateral sclerosis (ALS), are typically characterized by progressive neuronal loss and neurological dysfunctions in the nervous system, affecting both memory and motor functions. Neuregulins (NRGs) belong to the epidermal growth factor (EGF)-like family of extracellular ligands and they play an important role in the development, maintenance, and repair of both the central nervous system (CNS) and peripheral nervous system (PNS) through the ErbB signaling pathway. They also regulate multiple intercellular signal transduction and participate in a wide range of biological processes, such as differentiation, migration, and myelination. In this review article, we summarized research on the changes and roles of NRGs in neurodegenerative diseases, especially in AD. We elaborated on the structural features of each NRG subtype and roles of NRG/ErbB signaling networks in neurodegenerative diseases. We also discussed the therapeutic potential of NRGs in the symptom remission of neurodegenerative diseases, which may offer hope for advancing related treatment.

## Introduction

Neurodegeneration is characterized by the progressive loss of neuronal structure and function, including the death of neurons, which deteriorates over time and eventually leads to dysfunction of the nervous system. Neurodegenerative diseases, including Alzheimer’s disease (AD), Parkinson’s disease (PD), Huntington’s disease (HD), amyotrophic Ilateral sclerosis (ALS) and spinocerebellar ataxia (SCA; Mehta et al., 2016), are heterogeneous diseases typical to progressive deterioration of the structure and function of either the Central nervous system (CNS) or the Peripheral nervous system (PNS; Goedert and Spillantini, 2006; Masters et al., 2015), as the result of neurodegenerative processes (Mehta et al., 2016). According to different clinical and pathological characteristics, neurodegenerative diseases can be divided into acute and chronic neurodegenerative diseases (Gallucci et al., 2008). The former mainly includes cerebral ischemia (CI), brain injury (BI), and epilepsy, while the latter includes AD, PD, HD, and psychiatric disorders (Gallucci et al., 2008). The clinical manifestations of neurodegenerative diseases mainly include impairment of learning ability, cognitive ability, as well as the memory, sensory, judgments, thinking and motor abilities to varying extents (Kaplin and Montel, 2007).

As the human life expectancy prolongs with the improvement of modern medical management and living standards, the proportion of the elderly population is gradually increasing, and the population aging is acceleratively progressing (Boland and Mark, 2012). In the meantime, the incidence of neurodegenerative diseases is also increasing. According to a recent report, neurodegenerative diseases have become extremely serious threatening human health now a days. From the year 2010–2030, the number of individuals of age 65 years or above with PD will increase by 77%. For AD, the most common neurodegenerative condition, the number of individuals affected will increase by more than 50%. The increase in these and other neurodegenerative conditions will be accompanied by tremendous pressure and burden to patients, their families and even the government (Dorsey et al., 2013a, b). Although research on the mechanism and treatment of neurodegenerative diseases has developed rapidly in recent years, there are no effective medicines or drugs with high efficiency to prevent or treat these diseases (Hogan, 2014; Bateman, 2015). It is therefore a pressing challenge for neuroscientists to tackle the critical pathogenesis of neurodegenerative diseases and develop effective therapeutic means.

Accumulating evidence demonstrated that these neurodegenerative diseases exhibit similar changes at the subcellular level. Studies have found that oxidative stress (Bhat et al., 2015), mitochondrial dysfunction (Bhat et al., 2015), neuronal apoptosis (Okouchi et al., 2007), protein aggregation (Ross and Poirier, 2005) and autophagy (Ntsapi and Loos, 2016) are among the essential mechanisms underlying neurodegeneration and have been implicated in the progression of these diseases, including AD, PD and ALS.

Many causes have been reported to contribute to the development of neurodegenerative diseases, such as lack of nutrition provided to neurons or glial cells (Ntsapi and Loos, 2016), impaired axonal transmission (Patrizia et al., 2012) and metabolic pathways (Wang G.-X. et al., 2014; Spinedi and Cardinali, 2019), loss of energy due to neuronal mitochondrial damage (Bhat et al., 2015), protein misfolding (Khanam et al., 2016), neuroinflammation (Barnham et al., 2004), viral infections (Qiu et al., 2020), DNA mutations (Bao et al., 2006) and other factors. Researchers have found that many neurodegenerative diseases are characterized by protein misfolding and aggregation (Dobson, 2003; Wang X. et al., 2014; Lee et al., 2015; Lim and Yue, 2015), which are cytotoxic and can cause neuronal dysfunction. Amyloid plaques and neurofibrillary tangles (NFTs) were typically detected in the brain of AD patients (Selkoe, 1998). Lewy bodies formed by aggregation of α-synuclein were found in the PD brain (Costa et al., 2009). Insoluble macromolecular complexes due to mutations in genes encoding superoxide dismutase (SOD) were found to form aggregates, which ultimately leads to motor neuronal death (van der Graaff et al., 2009).

Recently, scientists have identified the structural characteristics of toxic polyglutamine proteins that lead to neurodegenerative brain diseases and the mechanisms of early neuropathology (Kwon et al., 2018). With the increase of human life expectancy, the proportion of the aging population increases, resulting in a gradually increasing number of people suffering from neurodegenerative diseases. Although many scholars are devoted to studying the pathogenesis and progression of neurodegenerative brain diseases, they have not yet uncovered the real cause. Due to the difficulty in diagnosing these diseases at the early stage, there is still too much work to be devoted to efficient treatment (Kwon et al., 2018). Researchers have found that the coiled-helix structure of toxic polyglutamine proteins, like a twisted telephone line in molecular structure, can cause rapid deformation of neurons and early neurodegenerative diseases, such as HD and spinocerebellar ataxia (Kwon et al., 2018). These proteins are abnormally combined to form a coiled-coil structure of toxic polyglutamine proteins. Researchers also found that the coiled-coil structure of toxic polyglutamine proteins in neurons can cause early neuropathy by binding to Foxo, a transcription factor that regulates the formation of dendrites (Kwon et al., 2018).

Neuregulins (NRGs) belong to the epidermal growth factor family of extracellular ligands, participating in the regulation of normal cells and tumor cell growth and survival through the ErbB family receptor tyrosine kinases (Holbro and Hynes, 2004; Bublil and Yarden, 2007; Schneider and Eckhard, 2010). The NRG family is mainly composed of NRG1–4 isoforms, among which NRG1 plays a vital role in regulating cardiac embryo development and maintaining cell structural function (Stavroula et al., 2003). Recent studies have shown that NRG1 can also antagonize inflammatory response, regulate cell growth, and inhibit cell apoptosis (Hedhli et al., 2014). However, few studies have focused on NRG2–4. These NRGs all contain an epidermal growth factor-like domain that regulates various biological processes primarily through the ErbB receptor signaling pathway (Schneider and Eckhard, 2010).

It is still unclear about the NRGs-involved mechanisms underlying the occurrence and pathogenesis of most neurodegenerative diseases. Most of the related studies are still in the exploratory stage, with a large amount of dispute about the role of the NRG family in neurodegenerative diseases. However, many studies have proved that the interaction between NRGs and their receptors plays an important role in the pathogenesis and treatment of neurodegenerative diseases (Bublil and Yarden, 2007). Therefore, dissecting the distribution and biological function of NRGs is one of the important entry points in studying the mechanism underlying neurodegenerative diseases. In addition, NRGs can be used as targets for therapeutic or clinical trials due to their multiple roles in these diseases. Future research should also consider NRGs treatment against neurodegenerative diseases, which may increase the success rate of the application of NRGs in related treatment. In this review article, we purposed to summarize the roles of NRGs in neurodegenerative diseases to provide some guidance to future research, which may to a certain degree offer hope for the clinical treatment of neurodegenerative diseases.

## Classification and Structures of Neuregulin

Neuregulin (NRG) is a family of growth factors that plays multiple roles in many neurological disorders, including ALS (Song et al., 2012), brain trauma (Pankonin et al., 2009), spinal cord injury (SCI; Pankonin et al., 2009), peripheral neuropathy (Calvo et al., 2010), and schizophrenia (Wang et al., 2015). There are four *NRG* genes that encode *NRG1*, *2*, *3* and *4*, respectively in mammals (Falls, 2003). All NRG isoforms contain a fragment encoding an epidermal growth factor (EGF)-like domain that regulates the association of NRG proteins with the ErbB receptor tyrosine kinases (Falls, 2003). It is known that the pleiotropic activities of NRGs are transduced by a set of receptor protein tyrosine kinases exhibiting structural similarity to the receptor for EGF (Gassmann and Lemke, 1997). Four transmembrane (TM) tyrosine kinases constitute the ErbB receptor family: Her1 (EGFR, ErbB1), Her2 (Neu, ErbB2), Her3 (ErbB3), and Her4 (ErbB4; Baulida et al., 1996). A large number of studies have shown the critical roles of NRGs and their receptors in cell proliferation regulation (Cespedes et al., 2018), neural crest cells differentiation and migration (Meyer et al., 1997), survival and maturation of astrocytes (Pinkas-Kramarski et al., 1994), Schwann cell development, maturation and myelination (Meyer and Birchmeier, 1995; Michailov et al., 2004; Fledrich et al., 2014; Bartus et al., 2016), growth and differentiation of epithelial, glial and muscle cells (Meyer et al., 1997), synapse formation (Fricker et al., 2011), GABAergic neuron migration (Yang et al., 1998), acute nerve injury (Fricker et al., 2011), chronic neuropathy (Fledrich et al., 2014, 2019), spinal cord injury (Bartus et al., 2016), cell fate determination (Baulida et al., 1996), and pattern establishment (Burden and Yarde, 1997) in the developing nervous systems.

### Structure, Distribution and Biological Functions of Neuregulin 1

**Figure 1:**
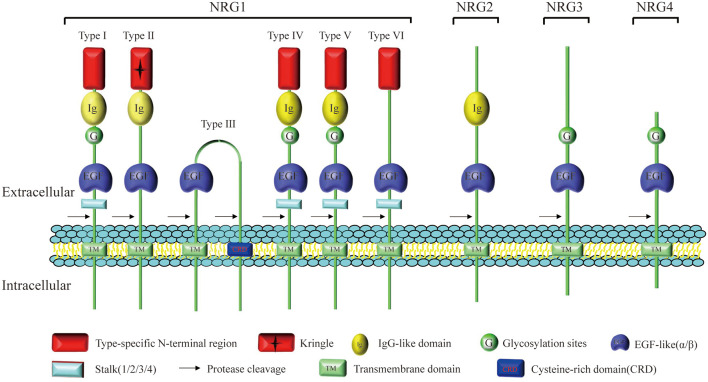
Structure and homology of neuregulin (NRG). All NRG isoforms contain a fragment encoding an epidermal growth factor (EGF)-like domain which regulates the association of NRG ligands with the ErbB receptor tyrosine kinase family. Different splicing of NRG1 gene can produce at least six NRG1 (I–VI) subtypes characterized by different extracellular domains, but only types I, II, IV, and V contain the Ig-like domain. Type II NRG1 contains a GGF-specific (“Kringle”) domain and type III NRG-1 contains a unique cysteine rich domain (CRD). NRG2 is most closely related to NRG1, and NRG1–3 exhibit high primary sequence homology, especially near the transmembrane (TM) domain. Compared with other NRGs containing multiple domains, such as the Ig-like domain, cysteine-rich domain (CRD) or mucin-like domain, NRG4 does not contain these recognizable domains except the EGF-like domain.

Neuregulin1 (NRG1) has been differently named as neu differentiation factor (NDF, type I), heregulin (HRG, type I), acetylcholine receptor-inducing activity (ARIA, type I), glial growth factor (GGF, type II), and sensory and motor neuron-derived factor (SMDF, type III; Kataria et al., 2019), which is the most widely studied among all NRG subtypes identified upto now. The NRG1 was originally extracted from breast cancer cells (Brockes, 1983), and was subsequently extracted from the bovine brain and pituitary gland (Lemke and Brockes, 1984). The *NRG1* gene is located on chromosome 8 of both human and mouse, and can encode 21 exons (Steinthorsdottir et al., 2004). At least 31 species can be generated by selective splicing and promoter differences, including soluble and membrane-tethered full-length subtypes (Falls, 2003). Differential splicing of NRG1 gene can produce at least six NRG1 (I–VI) subtypes with an extracellular domain, of which NRG1 type I to III are by far the most studied ones (Falls, 2003). All subtypes of NRG1 contain an EGF-like domain that can be further classified as α or β (Rosnack et al., 1994; Jacobsen et al., 1996). Different from soluble NRG1, the membrane-tethered isoform contains a transmembrane (TM) domain and an intracellular domain (ICD). The ICD can be further characterized as ICD a, b, and c. The structure that bridges the extracellular and TM domains is called stem (S) and can be further divided into S1, S2 and S4 (Zhao, 2013a). Proteolysis of NRG1 precursor proteins is a highly regulated biological process, releasing soluble domains to form autocrine/paracrine loops. However, the recruitment of S3 containing stop codon terminates the extension of the extracellular domain into the cytoplasm, leading to the formation of non-membrane anchored NRG1 α/β (Zhao, 2013a; Figure 1).

**Figure 2:**
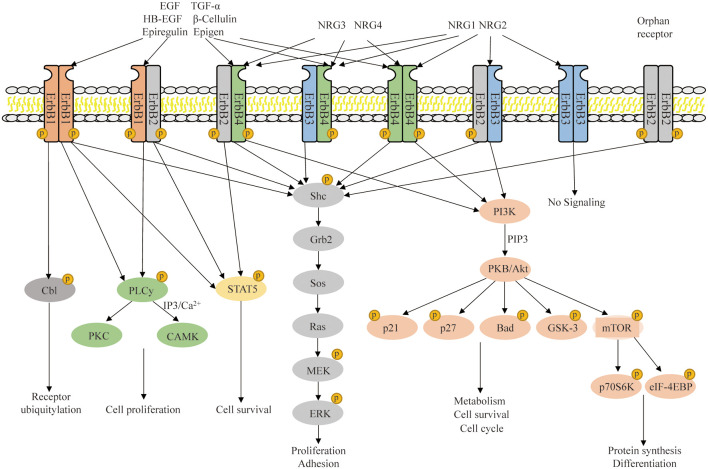
NRG/ErbB signaling and downstream signaling pathway. The NRG1–4 ligand interaction with ErbB1–4 increases their affinity and induces homodimerization and heterodimerization of ErbB1–4, thus activating the tyrosine kinase domain and allowing the phosphorylated activation of ErbB receptorsin the cytoplasmic region. These processes regulate multiple intercellular signal transduction and participate in a wide range of biological processes in the nervous system.

Researchers have found that all NRG1 subtypes contain an EGF-like domain essential for regulating biological activities. The NRG1 needs to bind to the dimerized ErbB receptors to transmit the signaling from the extracellular to the intracellular space. First, the EGF-like domain binds to the tyrosine kinase receptor ErbB3 or ErbB4, which subsequently forms a heterodimer with ErbB2, an orphan receptor lack of a ligand-binding domain, as a co-receptor in signal transduction (Carraway and Cantley, 1994). The ErbB3 lacks an activated tyrosine kinase domain and it heterodimerically binds to either ErbB2 or ErbB4 for signal transduction. The ErbB4 can form a homodimer by itself, but it preferentially forms a heterodimer with ErbB2. They are both co-expressed on the cell surface, and transmit extracellular signals to activate the intracellular signaling pathways, including phosphatidylinositol-kinase 3-kinase (PI3K)/protein kinase B (PKB/Akt), mitogen-activated protein kinase (MAPK)/extracellular signal regulated kinases (ERK) and p38/MAPK, thus being involved in a variety of both physiological and pathophysiological processes (Calvo et al., 2011; Figure 2).

**Figure 3:**
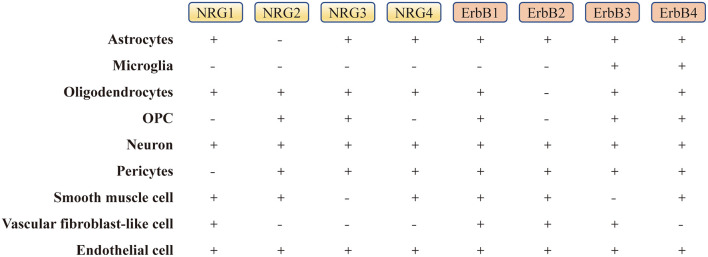
The expression level of NRGs and their ErbB receptors in the brain. “−” represents the absence or low level of expression, and “+” represents the presence or high level of expression in single cells (Zhang et al., 2014; He et al., 2018; Xu et al., 2018). OPC, oligodendrocyte precursor cell.

It has been proved that NRG1 precursors can be expressed predominately within cortical neurons, whereas soluble NRG1 accumulates preferentially on the surface of astrocytes of white matter. Research shows that NRG1 activity can also be detected in human cerebrospinal fluid, and its level seems to change in neuronal diseases. Although the level of NRG1 was found to be unchanged in the cerebrospinal fluid of patients with multiple sclerosis, it was found to be slightly reduced in that of ALS and PD patients, and significantly increased in that of AD patients (Pankonin et al., 2009). In addition, scientists have found that NRG1 is expressed in the gray matter, hypothalamus and cerebellum in the developing rat brain tissue. By comparison, NRG1 is abundantly expressed in developing brain tissues, including the hypothalamus, hippocampus, basal ganglia and brainstem (Bernstein et al., 2006; Figure 3). Some studies have shown that NRG1 and its receptors-ErbB4 are expressed in hypothalamic astrocytes, which can secret prostaglandin E2 under the stimulation of NRG1 in a paracrine manner, thus stimulating the release of luteinizing hormone releasing hormone (LHRH) and participating in sexual maturation (Bernstein et al., 2006). Recent studies have found that NRG1 can be expressed in pituitary gonadotrophin cells and phosphorylatively activate the prolactotroph surface receptors to promote the release of prolactin in a juxtacrine manner (Zhao and Ren, 2011a, b; Zhao et al., 2011); and *in vitro*, NRG1 can also promote the invasion of breast cancer cells, regulate the actin cytoskeleton, and promote cancer cell metastasis in both autocrine or paracrine manners (Hijazi et al., 2000; Miller et al., 2006). Furthermore, the interaction between NRG1 and its receptors can activate ERK, Akt, MAPK, PI3K, PKC and JAK-STAT signaling molecules, leading to cell cycle changes, cell differentiation, initiation of anti-apoptotic processes and tumorigenesis (Peles et al., 1993; Lydia et al., 2002; Table 1).

**Table 1:** Related binding receptors, major molecular function and biological process involved in neuregulins (NRGs).

Neuregulin	Receptor	Molecular function	Biological process
NRG1	ErbB3 ErbB4	Growth factor activity (Lee et al., 2001) Transmembrane receptor protein tyrosine kinase activator activity (Zhao et al., 1998); Transcription coregulator activity (Zhang and Hamburger, 2004); Cytokine activity (Osheroff et al., 1999); Chemorepellent activity (Gaudet et al., 2011); ErbB-2/3 class receptor binding (Ieguchi et al., 2010)	Negative regulation of transcription (Zhang and Hamburger, 2004); Negative regulation of neuron migration (Pan and Dobrowsky, 2013); Positive regulation of peptidyl-tyrosine autophosphorylation (Fleisig et al., 2004); Neurotransmitter receptor metabolic process (Rahimi-Aliabadi et al., 2017); Myelination in peripheral nervous system (Britsch, 2007); Glial cell differentiation (Kataria et al., 2019); Neural crest cell development (Britsch, 2007); Intracellular signal transduction (Gaudet et al., 2011); Activation of MAPK activity (Ieguchi et al., 2010); ErbB signaling pathway (Ieguchi et al., 2010); Cellular protein complex disassembly (Trinidad and Cohen, 2004)
NRG2	ErbB3 ErbB4	Growth factor activity (Lee et al., 2015); Transmembrane receptor protein tyrosine kinase activator activity (Hobbs et al., 2004); Signaling receptor binding (Chang et al., 1997)	ErbB2 signaling pathway (Hobbs et al., 2004); Nervous system development (Yarden, 2001); MAPK cascade (Brown and Sacks, 2009); Positive regulation of protein kinase B signaling (Burke et al., 2011); Intracellular signal transduction (Gaudet et al., 2011)
NRG3	ErbB4	Growth factor activity (Zhang et al., 1997); Transmembrane receptor protein tyrosine kinase activator activity (Zhang et al., 1997); Chemorepellent activity (Gaudet et al., 2011); Signaling receptor binding (Gaudet et al., 2011)	Intracellular signal transduction (Gaudet et al., 2011); Nervous system development (Yarden, 2001); Regulation of cell growth (Zhang et al., 1997); Modulation of chemical synaptic transmission (Zhou et al., 2018)
rtNRG4	ErbB4	Growth factor activity (Pfeifer, 2015); Transmembrane receptor protein tyrosine kinase activator activity (Schumacher et al., 2017); Signaling receptor binding (Harari et al., 1999); Protein binding (Luck et al., 2020)	Nervous system development (Buonanno and Fischbach, 2001); MAPK cascade (Brown and Sacks, 2009); Positive regulation of protein kinase B signaling (Mandelker et al., 2009); Regulation of cell motility (Marone et al., 2004)

### Structure, Distribution and Biological Functions of Neuregulin 2

**Figure 4:**
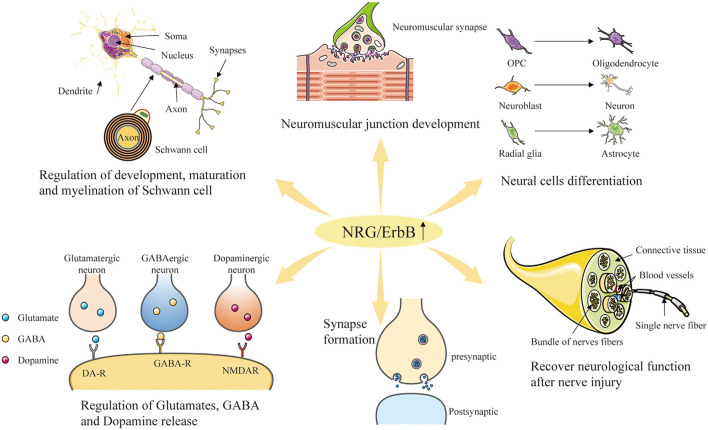
Diverse roles of NRG/ErbB signaling in the nervous system. Through the interaction with the ErbB-family receptors to increase phosphorylation on tyrosine residues, NRG family member proteins can induce the growth, proliferation and differentiation of epithelial, neuronal and glial cells, and potentially other types of cells. Furthermore, NRGs are involved in neuromuscular junction development, repair after nerve injury, synapse formation, and regulation of glutamate, GABA and dopamine release.

After the successful identification of *NRG1* gene, the other three genes, *NRG2* (Busfield et al., 1997; Carraway et al., 1997), *NRG3* (Zhang et al., 1997) and *NRG4* (Harari et al., 1999), which encode corresponding proteins, were successively found in later scientific studies. NRG2, also known as NTAK (neural-and thymus-derived activator for ErbB kinases), has been identified as the second member of the NRG family, with a similar structure to NRG1 (Busfield et al., 1997; Carraway et al., 1997; Figure 1). Although NRG1 and NRG2 are strongly expressed early in the developing brain, especially in the germinal layer, they are expressed in different brain tissue cell populations and distributed in different subcellular structures (Corfas et al., 1995; Longart et al., 2004). In the adult brain, the expression of NRG1 is generally decreased, scattered and widely distributed in the brain (Longart et al., 2004; Liu et al., 2011). In contrast, NRG2 is persistently expressed in three regions of the adult brain-cerebellum, olfactory bulb and hippocampal dentate gyrus (Carraway et al., 1997; Longart et al., 2004). Unlike NRG1 targeted to both axons and dendrites, NRG2 is concentrated in the dendrites of neurons (Longart et al., 2004; Figure 3). This unique temporal and spatial expression pattern of NRG2 suggests that it may have precised biological functions distinct from those of NRG1 (Yamada et al., 2000). Similar to NRG1, NRG2 transactivates ErbB1 and ErbB2 by directly binding to either ErbB3 or ErbB4, which can form a heterodimerized complex with them individually (Bublil and Yarden, 2007). Nakano et al. (2016) showed that NRG2 secreted by astrocytes can bind to ErbB3 receptor on neurons, promote neuronal survival and axon extension *in vitro*. This is similar to the role of NRG1 signaling pathway in glia-neuron interaction that promotes neuronal development, maturation and myelin formation (Fricker et al., 2011; Figure 4, Table 1).

### Structure, Distribution and Biological Functions of Neuregulin 3

*Neureguin3 (NRG3)* was originally found in DNA sequence databases and DNA libraries when screening for genes with sequence similarities to members of the NRG1 family (Zhang et al., 1997). As another member of the EGF family protein ligand, NRG3 has 31% homology with NRG1 in the amino acid sequence (Zhang et al., 1997). Some studies have found that the N-terminal domain of NRG3 is similar to SMDF (Ho et al., 1995), lacking Ig-like and spacer domains contained in most members of NRG1 family. Although some studies have predicted that the extracellular domain of NRG3 may lack common sites for N-linked glycosylation, some potential O-linked glycosylation sites have been found, and the similarity between the intracellular domain of NRG3 and that of NRG1 is very limited (Zhang et al., 1997; Figure 1). Scientists have proved that NRG3 is expressed in both embryonic and postnatal nervous systems (Zhang et al., 1997), including the important region adjacent to the rostral migratory stream (RMS; Stavroula et al., 2003; Anton et al., 2004). As a specific ligand of receptor tyrosine kinase ErbB4, the structure and polymorphism of NRG3 are associated with neurodevelopmental disorders, including developmental retardation, cognitive impairment, autism and schizophrenia (Meier et al., 2013). Intriguingly, however, NRG3 can also bind to ErbB4, thus synergistically affecting proliferation, migration and differentiation of neuroblasts as a paralog of NRG1 (Table 1).

### Structure, Distribution and Biological Functions of Neuregulin 4

Neuregulin4 (NRG4) was originally detected in adult pancreas and muscle and was initially identified as a necessary factor for tissue growth (Harari et al., 1999). Further studies have shown that NRG4 is also present in human breast milk and growing intestinal tissue, where it promotes the survival of epithelial cells and prevents the necrosis of intestinal cells (Bernard et al., 2012; McElroy et al., 2014). In addition, studies have shown that NRG4 is highly expressed in prostate cancer, breast cancer, and gastric cancer (Hayes et al., 2010; Mariana et al., 2010; Nielsen et al., 2014).

As a homologous ligand of the EGF/NRG family, NRG4 has very low sequence homology with known members of the NRG family besides the EGF-like domain (Harari et al., 1999). In contrast, the other three NRGs (NRG1–3) exhibit high primary sequence homology especially near the transmembrane domain (Harari et al., 1999). It has been found that the precursor form of NRG4 has the same structural characteristics as an ErbB ligand in mammals (Massagué and Pandiella, 1993), including the transmembrane domain, EGF-like domain proximal to the membrane, and the proteolytic cleavage site in the serine-rich region at the C-terminus of EGF-like domain (Harari et al., 1999). Besides the two potential N-linked glycosylation sites in the known extracellular domain of NRG4, the ectodomain region also contains the O-linked glycosylation site (Harari et al., 1999). Although NRG4 lacks hydrophobic signal peptide for N-terminal localization, like other NRGs, it is different from the most EGFR specific ligands (Falls, 2003; Figure 1). However, the absence of these sequence characteristics does not rule out the possibility that NRG4 can be secreted as a growth factor, in that, other growth factors without a signal peptide, can be secreted or released from cells by alternative secretion mechanisms or autolysis (Burden and Yarde, 1997). In addition, NRG4 lacks non-polar amino acids (Harari et al., 1999), which usually replace the signal peptide. And the hypothetical extracellular domain of NRG4 is the shortest among members of the NRG/EGF family (Harari et al., 1999). Unlike other NRGs, the NRG4 does not contain the recognizable domains, such as the Ig-like domain, cysteine-rich domain (CRD) and mucin-like domain, except the EGF-like domain (Harari et al., 1999; Table 1).

## Neuregulins in The Development and Therapeutics of Neurodegenerative Diseases

### Neuregulins and AD

Alzheimer’s disease (AD) is a neurodegenerative disease with insidious and progressive onset, mainly in the elderly, and its incidence increases with age (Masters et al., 2015). As a disease entity, AD shares many characteristics with other molecularly defined neurodegenerative diseases including PD and frontotemporal dementia (FTD). It is therefore deduced that AD is the result of abnormal aging, which increases the risk of AD. In the most clinical aspects, the sporadic and familial forms of AD are comparable, including the rate of disease progression and biomarker profiles (Chang et al., 2016). A recent study (Talboom et al., 2019) found that memory loss may indicate an early sign of a severe problem in a young person with a family history of AD.

Alzheimer’s disease (AD) is a continuously progressive disease clinically characterized by changes in cognition or behavior (Gallucci et al., 2008; Masters et al., 2015). First, mild symptoms are mainly caused by memory loss, but life can still be maintained by self-care. Second, moderate symptoms are characterized by memory decline, obvious cognitive defects, and disorientation in time and place, and the patients have to be supported to take care of their life. Finally, severe symptoms are characterized by severe mental decline, and the patients are unable to carry out outdoor activities independently and they cannot take care of their life themselves. During the progression of the disease, the patients may also experience changes in mood and personality. They may experience abnormal mood swings, such as agitation, irritability, impulsivity, lack of motivation, apathy and paralysis (Goedert and Spillantini, 2006).

Alzheimer’s disease (AD) is pathologically characterized mainly by: (1) senile plaque formed by abnormal deposition of β-amyloid protein outside neurons; and (2) NFTs formed by abnormal phosphorylation of tau protein (Masters et al., 2015). Recent studies have shown that abnormal phosphorylation of tau protein is more highly correlated with AD than abnormal deposition of β-amyloid protein. A large amount of abnormally aggregated tau protein exists in the brain of AD patients. Abnormal modification and content change of tau protein play an important role in the clinicopathogenesis of AD (Bussian et al., 2018).

According to the 2018 Alzheimer’s Clinical Trials Report conducted by Alzheimer’s Drug Discovery Foundation (ADDF) (Alzheimer’s Drug Discovery Foundation, 2019), the drug treatment effect on AD is very limited, while research on pharmacology has not yet developed effective drugs to treat AD, only offering symptomatic relief. Currently, the pathogenesis of AD is not clear due to the complicated cause of the disease, which has comprised the main dilemma of new drug research and development. Regarding the cause of AD, the mainstream view is still a series of insults caused by abnormal aggregation of β-amyloid (Aβ) and hyperphosphorylation of tau protein (Grundke-Iqbal et al., 1986; Arriagada et al., 1992; Carson and Turner, 2002). Scientists have carried out many drug screening tests, animal trials, clinical research and other experimental work targeting these two major pathogenesis mechanisms. In the past 20 years, drug research and development have focused on clearance of the Aβ plaques in the brain (Morimoto, 2010), but most of the alternatives have not been successful. The therapeutic effect of available drugs against AD is relatively limited, and there is no efficient way to stop the progression of the disease (Masters et al., 2015). At present, the anti-AD drugs used clinically are medically attributed to “neurotransmitter therapeutic drugs,” and the effects are obtained by changing the concentration or intensity of neurotransmitters in the brain of AD patients (Hara et al., 2019). For instance, the mainstream anti-AD drug, cholinesterase inhibitor, can increase in the brain the concentration of acetylcholine and improve cognition, whose absence has been considered a potential cause of AD.

### Neuregulin 1 and AD

As cell-to-cell signaling proteins that are ligands for receptor tyrosine kinases of the ErbB receptor family (Falls, 2003), NRGs and their corresponding receptors play pivotal roles in organ development and maintenance, as well as in many disorders (Gallucci et al., 2008; Cespedes et al., 2018; Yan et al., 2018; Kataria et al., 2019). The NRG1 has been originally reported to be associated with schizophrenia (Harrison and Law, 2006) and has been implicated in neuro developmental processes including neuronal differentiation and synapse formation (Buonanno and Fischbach, 2001). NRG1 can promote neurite outgrowth in hippocampal and thalamic primary neurons (Gerecke et al., 2004), and even can attenuate cognitive function impairments in AD mice (Ryu et al., 2016). Jiang et al. (2016) suggested that NRG1 may serve as a preventive agentby influencing the pathological development of AD. Woo et al. (2011) discovered that both ErbB4 and phospho-ErbB4 immunoreactive intensities were higher in neurons of the CA1–2 transitional field of AD brains as compared to age-matched normal controls. ErbB4 expression was increased in the neurons of the corticomedial amygdala nucleus, human basal forebrain, as well as those of the superior frontal gyrus of AD. In the cerebral cortex and hippocampus of amyloid precursor protein (APP)/presenilin 1 (APP/PS1) double transgenic mice, ErbB4 immunoreactivity is significantly increased compared with the age-matched wild type control. These combined reports indicated that the abnormal change of ErbB4 may be involved in the progression of AD pathology and NRG1 can undertake neuroprotective actions against Swedish amyloid precursor protein-induced neurotoxicity (Woo et al., 2011; Table 2).

**Table 2:** Differential expression of NRGs and their ErbB receptors in Alzheimer’s disease (AD) brain (vs. normal brain; Xu et al., 2018; Zhang et al., 2019).

	NRG1	NRG2	NRG3	NRG4	ERBB1	ERBB2	ERBB3	ERBB4
Entorhinal cortex	*	NA	***	ns	***	***	**	*
Hippocampus	ns	ns	*	ns	**	ns	ns	ns
Temporal cortex	*	*	NA	ns	*	ns	*	ns
Frontal cortex	ns	**	NA	NA	ns	ns	*	ns

ns, *P*-value > 0.05; *P-value < 0.05; **P-value < 0.01; ***P-value < 0.001; NA, not available.

In addition to the Aβ peptide, other metabolites of amyloid precursor protein (APP) such as the C-terminal fragments of APP (APP-CTs) have been reported to exert cytotoxic effects in neuronal cells (Baik et al., 2016). Ryu et al. (2012) investigated whether NRG1 exerts neuroprotective effects against APP-CTs and attempted to determine the underlying neuroprotective mechanisms. Their results showed that NRG1 alleviates the neurotoxicity induced by the expression of APP-CTs in neuronal cells. NRG1 also reduces the accumulation of reactive oxygen species and attenuates mitochondrial membrane potential loss induced by APP-CTs, which can be blocked by ErbB4 inhibition (Ryu et al., 2012).

**Figure 5:**
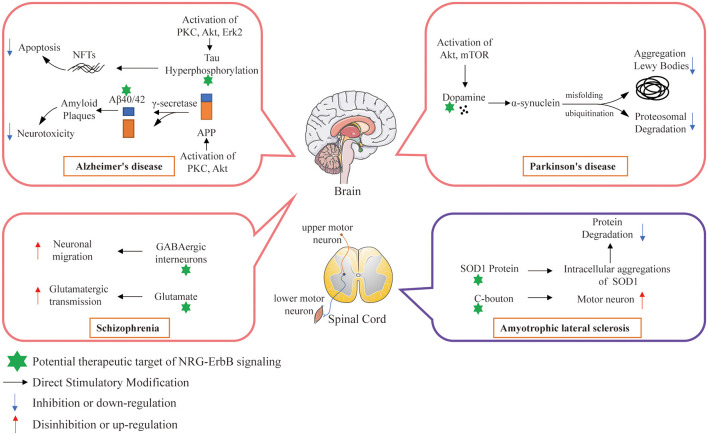
Potential therapeutic targets of NRG/ErbB signaling involved in selected neurodegenerative diseases.

NRG1 exerts pivotal functions in both brain development and plasticity, as well as in neuroprotection (Ryu et al., 2012; Willem, 2016; Xu et al., 2017). Another research (Baik et al., 2016) focused on the downstream signaling pathways of NRG1 and their roles in the prevention of Aβ42-induced neurotoxicity, demonstrating that inhibition of PI3K/Akt activation abolished the ability of NRG1 to prevent Aβ42-induced LDH release and increase the counting numbers of TUNEL-positive cells and reactive oxygen species (ROS) accumulation in primary cortical neurons. These studies further confirm that NRG1 signaling exerts a neuroprotective effect against Aβ42-induced neurotoxicity *via* activation of the PI3K/Akt signaling pathway, with a neuroprotective potential for the treatment of AD (Baik et al., 2016; Figure 5).

In the nervous system, the functions of NRG1 are essential for peripheral myelination, the establishment and maintenance of neuromuscular and sensorimotor systems and the plasticity of cortical neuronal circuits (Cespedes et al., 2018). It has been reported that intracerebroventricular infusion of NRG1 attenuated cognitive impairments in a 13-month-old Tg2576 AD mouse model (Ryu et al., 2016). In addition, NRG1 can rescue the reduction in the Golgi-Cox staining-based counting number of dendritic spines in the brain of Tg2576 mice (Ryu et al., 2016). The NRG1 also alleviated the decrease in neural differentiation induced by oligomeric Aβ1–42 in mouse fetal neural stem cells with therapeutic potential for AD by alleviating the reduction in dendritic spine density and neurogenesis found in the AD brain (Ryu et al., 2016). The NRG1 regulates neuronal survival, synaptogenesis, astrocytic differentiation, and microglial activation, thus being involved in the development of the nervous system. Research has demonstrated the expression and distribution of NRG1 and ErbB kinases in the hippocampus of AD patients and in transgenic mice co-expressing AD-associated mutations of β amyloid precursor protein (APPK670N, M671L) and presenilin-1 (PS1M146L; Chaudhury et al., 2003). NRG1 and its three ErbB receptors (ErbB2, ErbB3 and ErbB4) are expressed in distinct cellular compartments of neurons in the hippocampi of both control humans and wild type mice. In the AD brain, upregulated NRG1 was found in both microglia, and to a less extent dystrophic neurites in neuritic plaques, suggesting that both autocrine and paracrine interactions can regulate the action of NRG1 within these lesions. It can thus be inferred that NRG1 and ErbB4, as well as ErbB2, are similarly associated with neuritic plaques in the APP/PS1 transgenic mice (Chaudhury et al., 2003; Table 3).

**Table 3:** Convergent functional genomic (CFG) ranking for NRGs and their ErbB receptors in AD (Xu et al., 2018; Zhang et al., 2019).

	eQTL	GWAS	PPI	Pathology		CFG
				Aβ	Tau	
NRG1	2	212	PSEN1, APOE	−0.118 ns	−0.614*	5
NRG2	3	7	PSEN1	NA	NA	3
NRG3	3	1	PSEN1	−0.378*	−0.357^ns^	4
NRG4	2	0	PSEN1	0.002^ns^	−0.106^ns^	2
ErbB1	2	2	PSEN1, PSEN2, APOE	0.119^ns^	0.012^ns^	3
ErbB2	3	0	PSEN1, PSEN2, MAPT	NA	NA	2
ErbB3	1	0	APP, PSEN1, MAPT	NA	NA	2
ErbB4	4	0	PSEN1, PSEN2	NA	NA	2

eQTL: expression of target gene is regulated by AD genetic variants (genetic variants: IGAP GWAS *P*-value < 1E-3; eQTL: *P*-value < 1E-3); GWAS: IGAP *P*-value < 1E-3; PPI: target gene has significant physical interaction with APP, PSEN1, PSEN2, APOE or MAPT (*P*-value < 0.05); Pathology (Aβ): correlation of target gene expression with AD pathology in Aβ line AD mouse models (*r*, *P*-value; ^ns^*P*-value > 0.05; *P-value < 0.05; NA, not available; Pathology (Tau): correlation of target gene expression with AD pathology in tau line AD mouse models (*r*, *P*-value; ^ns^*P*-value > 0.05; *P-value <0.05; NA, not available; CFG: total CFG score of target gene. One CFG point is added if any of above evidence is significant, CFG point ranges from zero to five. CFG ranks is an approach integrating multiple lines of evidence, for example, genetic association, PPI, gene expression alteration, and pathology correlation, to prioritize AD candidate genes.

Inhibition of the protease β-site amyloid precursor protein-cleaving enzyme 1 (BACE1) represents a potential treatment for AD, and lots of BACE inhibitors are progressing through clinical trials at present (Munro et al., 2016). BACE was first identified through its function as the rate limiting enzyme of amyloid-β peptide (Aβ) production. Scientists have aimed to reduce the production of amyloid-β (Aβ) peptide from the amyloid precursor protein (APP) to reduce or prevent Aβ toxicity (Ryu et al., 2012; Masters et al., 2015; Cespedes et al., 2018). Over the last decade, it has become clear that BACE1 proteolytically cleaves several substrates in addition to APP. These substrates are not only known to be involved in the pathogenesis of AD, but have other roles in the developing and/or mature CNS (Munro et al., 2016). Among BACE1 substrates that have been validated to date, some may contribute to the synaptic deficits resulting from BACE blockade, including NRG1, close homolog of L1 and seizure-related proteins (Munro et al., 2016; Chen et al., 2017). NRG1 was identified as a major physiological substrate of BACE1 during early postnatal development when phenotyping similarities between BACE1 KO mice and NRG1 heterozygous mice. Due to its hairpin nature, type IIINRG1 is subjected to a secondary cleavage by BACE1 to release the EGF-like domain on the basis of the initial proteolytic cleavage (Willem, 2016). Thus, it is important to understand the possible role of blocking the effects of BACE on NRG1, as well as other proteins detected in substrate screening, and to develop substrate-selective BACE inhibitors against AD.

Some recent research suggests that NRG1 signaling is of great importance in cognitive function and neuropathology in AD (Ryu et al., 2016; Cespedes et al., 2018; Talboom et al., 2019). Xu et al. (2016) overexpressed full-length type I or type III NRG1 *via* lentiviral vectors in the hippocampus of line 41 AD mouse. They found that both type I and type III NRG1 can improve memory deficits scored by the Morris water-maze behavioral task, accompanied by neuropathological ameliorations (Xu et al., 2016). Consistent with the result of increased neprilysin (NEP) immunoreactivity in the hippocampus of AD mice, the study also showed that soluble ectodomains of type I and type III NRG1s significantly increased expression of Aβ-degrading enzyme NEP in primary neuronal cultures (Xu et al., 2016). Furthermore, Chang et al. (2016) found that plasma soluble NRG1 (sNRG1) is among the biomarkers for AD diagnosis. They investigated a potential difference in the level of the plasma proteins between 60 AD patients and 55 healthy subjects. Their research showed that the level of sNRG1 was significantly higher both in mild AD and in moderate AD patients compared with that of the normal control subjects (Chang et al., 2016).

Although ErbB4 is expressed in multiple regions of the adult animal brain, findings on its role in AD are still limited. Research shows that ErbB4 immunoreactivity is significantly increased in apoptotic hippocampal pyramidal neurons in the brain of AD patients, where it is co-localized with the apoptotic signaling molecule Bax. Thisobservation suggests that up-regulation of ErbB4 immunoreactivity in apoptotic neurons may involve in the pathological progression of AD (Woo et al., 2011). In the brain, from both AD patients and that from the APP/PS1 transgenic AD mouse, ErbB4 can be expressed by reactive astrocytes and microglia surrounding the neuritic plaques. In AD brains, NRG1 is up-regulated in microglia, dystrophic neurites, and in neuritic plaques, indicating that NRG1-based autocrine and/or paracrine interactions exist within these lesions (Chaudhury et al., 2003).

NRG1, ErbB4 and ErbB2 are similarly associated with neuritic plaques in the APP/PS1 transgenic AD mouse. It has been reported that the hippocampal distribution of NRG1 and ErbB4 was altered in AD (Chaudhury et al., 2003; Table 2). Also, scientists inactivated ErbB2/ErbB4-mediated NRG1 signaling specifically in the CNS by CRE/LOX technology (Barros et al., 2009). Although they found that cortical layers develop normally in the cerebral cortex, hippocampus, and cerebellum, the loss of ErbB2/ErbB4 functions influences dendritic spine maturation and disturbs the interaction between postsynaptic scaffold proteins and glutamate receptors, and ErbB2/ErbB4 receptors-deficient mice exhibit enhanced aggression and lower prepulse inhibition (Barros et al., 2009). Further research found that in APP/PS1 transgenic mice, NRG1β1 antagonizes neuronal apoptosis *via* ErbB4-dependent activation of PI3-kinase/Akt. The extracellular domain of NRG1β1 (NRG1β1-ECD) increase the levels of pErbB4 receptor and pAkt, and increase the level of Bcl-2 both in *in vitro* studies and APP/PS1 transgenic mice (Cui et al., 2013). Riethmacher et al. (1997) found that both sensory and motor neurons require factors like CNTF, GDNF, BDNF, LIF, PDFG, FGF, NT-3 or TGF-β for survival, development, maintenance and regeneration provided by developing Schwann cells. Taveggia et al. (2005) found ensheathment fate of axons can be determined by NRG1 type III, which is the sole NRG1 isoform retained at the axon surface to activates PI3-kinase for Schwann cell myelination. Meanwhile, in ErbB3 mutant embryos, most motor neurons and sensory neurons in dorsal root ganglia undergo cell death at later stages, indicating that ErbB3 functions in a cell-autonomous way during the development of Schwann cells, but not in the survival of sensory or motor neurons (Riethmacher et al., 1997; Figure 5).

### Neuregulin 2 and AD

As a member of the epidermal growth factor (EGF) family, NRG2 can bind directly to ErbB3 and ErbB4, thus transactivating ErbB2 (Nakano et al., 2004). Although research has demonstrated that NRG1 can promote axonal regeneration and play a neuroprotective role in neurodegenerative diseases (Ryu et al., 2012; Mancuso et al., 2016; Xu et al., 2016), it remains unclear about the biological functions and related mechanisms of NRG2 in neurodegenerative diseases, especially in AD. Researchers observed that NRG2β is a potent agonist for ErbB4 receptor. However, NRG2α, which is derived from the splicing isoform of the same gene also encoding NRG2β, is an inefficient ErbB4 agonist (Hobbs et al., 2004). Studies showed that the substitution of a lysine residue for Phe45 in NRG2β results in a reduced ligand potency, and substitution of a phenylalanine for Lys45 in NRG2α enhances the ligand potency (Hobbs et al., 2004).

Nakano et al. (2016) showed that NRG2 secreted by astrocytes binds to ErbB3 receptor on neurons and promotes neuronal survival and axonal extension *in vitro*, which is similar to the role of NRG1 in the CNS development, maturation and myelin formation of glial-neuron interactions. After analysis of the regional and temporal expression of NRG1–3 and comparison of the processing and subcellular distribution of NRG2 with NRG1 proteins, Longart et al. (2004) found that NRG2 is developmentally regulated and targeted to dendrites of central neurons. To further investigate the roles of NRG2 in synaptogenesis and dendritic growth, Lee et al. (2015) depleted NRG2 at different developmental stages of newborn GCs with a tetracycline-inducible expression system, showing that dual roles of NRG2 in the newborn neurons. The extracellular domain of NRG2 mediates synaptogenesis by binding ErbB4 receptor on GABAergic neurons *via* forward signaling pathways. In contrast, the intracellular domain of NRG2 contributes to dendritic outgrowth and glutamatergic synapse maturation *via* reverse signaling pathways (Lee et al., 2015). Vullhorst et al. (2015) also confirmed that NRG2 can bi-directionally bind NMDA and ErbB4 receptors in the cortical GABAergic interneurons involved in glutamatergic transmission. Considering that neuronal development is a critical process of brain development, adult neurogenesis enhancement by NRG2 may represent a therapeutic strategy against both age and disease-related neuronal loss.Although there is no direct evidence to prove the effect of NRG2 in the treatment of AD, its potential cannot be ignored.

### Neuregulin 3 and AD

Neuregulin (NRG3), a specific ligand for ErbB4 and a neuronal-enriched neurotrophin, has been identified as a protein structurally related to the NRG1. It is involved in the genetic predisposition to a broad spectrum of neurodevelopmental, neurocognitive and neuropsychiatric disorders, including AD, autism and schizophrenia (Gallucci et al., 2008; Paterson and Law, 2014; Wang K.-S. et al., 2014; Table 4). It plays an important role in neuronal development, including plasticity, development, differentiation, and proliferation (Zhou et al., 2020).

**Table 4:** Overview of selected neurodegenerative diseases.

Diseases	Clinical characteristics	Neuropathology	Lesion site	Protein	Associated NRGs
Alzheimer’s disease	Cognitive and memory impairment	Neurofibrillary tangles (NFTs); Amyloid plaques	Hippocampus; Cerebral cortex	Microtubule-associated proteintau (tau); Amyloid-β (Aβ)	NRG1; NRG3
Parkinson’s disease	Rest tremor; Rigidity; Bradykinesia	Damage of dopaminergic neurons; Formation of Lewy bodies	Substantia nigra; Hypothalamus	α-Synuclein	NRG1
Amyotrophic lateral sclerosis	Dysphagia; Partial muscle stiffness; Dyskinesia	Upper and lower motor neuron loss; Bunina bodies; Astrocytic hyaline inclusions	Motor cortex; Brain stem	Superoxide dismutase 1 (SOD1)	NRG1
Schizophrenia	Perception, thinking, emotion and behavior disorientations	Dyssecretion of dopamine and 5-hydroxytryptamine	Cerebral cortex	Dysbindin; Ubiquitin	NRG1; NRG2 NRG3; NRG4

The genetic studies have suggested that ErbB4 activation may also be regulated in the CNS by NRG3 (Zhang et al., 1997). Although knowledge of the genetic involvement of NRG3 in neurological diseases has been vastly expanded, little is known about its roles in the risk mechanisms of the development of neurodegenerative diseases (Meier et al., 2013; Loos et al., 2014; Wang K.-S. et al., 2014). Paterson and Law (2014) found that developmental overexposure to NRG1 induced multiple non-CNS mediated peripheral effects and severely disrupted the performance of prepulse inhibition of the startle response. In contrast, NRG3 exerts no effects on any peripheral measures investigated or on sensorimotor gating. In particular, developmental NRG3 overexposure even produced an anxiogenic-like phenotype and deficits in social behavior in adulthood, indicating that NRG3 plays an important role in brain development and function, appearing to be distinct from NRG1 (Paterson and Law, 2014).

*NRG3* gene at 10q22–q24 has been implicated in multiple psychiatric traits such as cognitive impairment (Meier et al., 2013). To discover the role of the *NRG3* gene polymorphism in AD, scientists explored the relationship between *NRG3* and age-of-onset of AD (AAO) and the risk of AD development (Wang K.-S. et al., 2014). By logistic regression and linear regression analyses, secondary data analysis of 257 single-nucleotide polymorphisms (SNPs) in *NRG3* was performed in 806 AD patients and 782 controls. Using an independent family-based sample, scientists found one SNP rs11192423 associated with AAO both in the case-control sample (*p* = 0.0155) and in the family sample (*p* = 0.0166), indicating that genetic variants in the *NRG3* gene play a role in AD and SNPs in the *NRG3* genes and were more strongly associated with AAO of AD (Wang K.-S. et al., 2014).

Recent research found that NRG3 and ErbB4 are located at presynaptic and postsynaptic structures, respectively, in excitatory synapses on parvalbumin-positive interneurons. And the ablation of NRG3 can lead to a similar phenotype to that of ErbB4 ablation, including reduced excitatory synapse numbers on parvalbumin-positive interneurons, altered short-term plasticity, and disinhibition of the hippocampal network. Further investigation demonstrated that presynaptic NRG3 increases excitatory synapse numbers and affects short-term plasticity of ErbB4-positive interneurons. These results indicate that NRG3 can promote excitatory synapse formation of hippocampal interneurons and can play a crucial role in synaptogenesis and synapse functions (Müller et al., 2018).

### Neuregulin 4 and AD

Studies have shown that the extracellular fragment with the EGF-like domain produced by NRG4 proteolysis usually acts on target cells to exert their functions in the form of autocrine, paracrine or juxtacrine manner (Hayes et al., 2010; Mariana et al., 2010; Nielsen et al., 2014). In the meantime, more and more research has found that NRG4 plays an important role in controlling energy balance, increasing insulin sensitivity and reducing the development of fatty liver (Wang G.-X. et al., 2014; Dai et al., 2015).

Through analysis of NRG4-deficient mice, Paramo et al. (2018) found that both the apical and basal dendrites of neocortical pyramidal neurons are significantly stunted in NRG4−/− neonates *in vivo* compared to NRG4+/+ littermates, and recombinant NRG4 rescued the stunted phenotype of embryonic neocortical pyramidal neurons cultured from NRG4−/− mice. However, they observed that the pyramidal dendrite arbors of NRG4−/− and NRG4+/+ mice were similar in the adult, demonstrating compensatory changes that occur in the pyramidal dendrites of NRG4−/− mice with age. The majority of cultured NRG4+/+ mice embryonic cortical pyramidal neurons co-express NRG4 and its receptor ErbB4 (Paramo et al., 2018). These results show that NRG4 serves as a major physiologically relevant regulator of the growth and elaboration of pyramidal neuronal dendrites in the developing neocortex. Meanwhile, Paramo et al. (2019) found that NRG4 can also function as a significantly novel regulator of dendritic growth and arborization and spine formation in the striatum, exerting its effects by an autocrine/paracrine mechanism (Paramo et al., 2019). Meanwhile, NRG4 can act directly on striatal medium spiny neurons during development *in vivo* (Paramo et al., 2019). These studies showed that NRG4 has a crucial function in the developing brain. Thus, it will be interesting to ascertain how the putative NRG4/ErbB4 autocrine loop is regulated in pyramidal neurons and investigate how NRG4 contributes to the pathogenesis of particular neurodegenerative diseases such as AD.

## Neuregulins in Other Neurodegenerative Diseases

### Neuregulins in Parkinson’s Disease (PD)

Parkinson’s disease (PD) follows AD as the second severe neurodegenerative disease (Reitz et al., 2011), with morbidity of more than 1% in people above the age of 60 years, 5% in people above the age of 65 years, and 20% in people above the age of 80 years old (Bloodsworth et al., 2000; Przedborski, 2017). The clinical manifestations of PD are mainly characterized by resting muscle tremors, myotonia, retardation of movement, postural reflex disorder, and other motor neuronal dysfunctions (Li et al., 2017; Przedborski, 2017). In addition, some patients also suffer from abnormal autonomic nervous system functions, such as cognitive impairment, autonomic nerve dysfunctions and etc. (Caligiore et al., 2016; Malek et al., 2017). The main pathological features of PD patients were the damage and loss of dopaminergic neurons in the compact part of substantia nigra (SN), the decrease of dopamine levels in the striatum and the formation of Lewy bodies in the cytoplasm of residual neurons (Irene et al., 2012; Table 4). Studies have shown that the decrease of striatal dopamine levels can lead to the decrease of dopamine innervations in the SN-striatal pathway, the relative enhancement of cholinergic nerve functions and the occurrence of motor dysfunctions in PD patients (Irene et al., 2012; Figure 5).

At present, it is believed that the etiology of PD is mainly related to age, environment and genetic factors (Caligiore et al., 2016). In the meantime, more than ten PD-related genes have been found, among which the most common ones are LRRK2, α-synuclein, PINK1, DJ-1, Parkin and others (Chou and Kah-Leong, 2013), thus providing some basis for further studying the molecular mechanism of PD. Despite intensive drug development, symptomatic treatment is still the main therapeutic method clinically. The commonly used drugs include dopamine analogs and anticholinergics, but there is no specific cure for PD.

In the past decade, researchers came to pay attention to the role of NRGs in PD, to identify strategies to cure or alleviate PD. Carlsson et al. (2011) studied the effect of systemic administration of NRG1β1 on dopaminergic neurons in a mouse model of PD, and they found that dopamine levels were increased in SN and striatum of the treated adult mouse. NRG1β1-ECD also significantly protected the mouse nigrostriatal dopaminergic system against 6-hydroxydopamine (6-OHDA)-induced toxicity both morphologically and functionally *in vivo*, and protected human dopaminergic neurons against 6-OHDA *in vitro*. In another 1-methyl-4-phenyl-1,2,3,6-tetrahydropyridine (MPTP) toxin-based mouse model of PD, researchers also found that systematic administration of NRG1β1-ECD can rescue nigral dopaminergic neurons *via* the ErbB4 receptor tyrosine kinase (Depboylu et al., 2015). However, NRG1β1-ECD cannot reverse MPTP-induced decrease in dopamine levels and dopaminergic fibers in the striatum. In addition, NRG1β1-ECD cannot affect the conversion of MPTP to its toxic metabolite 1-methyl-4-phenylpyridinium (MPP) as well as levels of the dopamine transporter to influence the intracellular uptake of MPP (Depboylu et al., 2015). In addition, they also found that ErbB4 is upregulated in midbrain dopaminergic neurons in PD, which may reflect a better survival of ErbB4-positive neurons or an increased expression of ErbB4 by residual neurons to pursue neurotrophic support (Depboylu et al., 2013). Hama et al. (2015) found that the level of plasma type III NRG1 is reduced and is specifically associated with idiopathic PD, although its correlation with the clinical severity of PD has not been confirmed. Thus, NRG1 type III reduction may facilitate the etiological diagnosis of PD.

### Neuregulins in Amyotrophic Lateral Sclerosis (ALS)

Amyotrophic lateral sclerosis (ALS), also known as motor neuron disease (MND) or Lou Gehrig’s disease, is a disease that causes the death of neurons controlling voluntary muscles (Lee and Kim, 2015). It is characterized by the degeneration of both upper and lower motor neurons and leads to muscle weakness and eventual paralysis (Hardiman et al., 2017). Except for the familial/genetic susceptibility, the etiology of ALS remains unknown in most patients. Although the main symptoms of ALS are related to motor dysfunction (such as muscle weakness, cramps, and dysphagia), upto 50% of patients experience cognitive and/or behavioral disorders during the disease procession. 13% of patients present with concomitant behavioral variant prontotemporal dementia (FTD; Neumann et al., 2006; Phukan et al., 2012; Elamin et al., 2013), which contributes to the recharacterization of ALS as a neurodegenerative rather than a neuromuscular disorder and points out the direction of future research (Table 4).

In a recent study on the relationship between ErbB4 and ALS/FTD, ErbB4 mutation was found for the first time in ALS/FTD and that its mutation reduced auto-phosphorylation of upon NRG1 stimulation (Sun et al., 2020). Song et al. (2012) analyzed the relationship between NRG1 isoform expression and glial cell activation and motor neuron loss in the spinal cord of ALS patients during the disease progression in a superoxide dismutase 1 (SOD1)-mutated ALS mouse model. They observed microgliosis, astrocytosis, and motor neuron loss in the ventral horn of ALS patients, and these symptoms increased as the disease progressed in the model. The expression of membrane-tethered type III NRG1 decreased in parallel with the motor neuronal loss, while secretory type I NRG1 expression increased, which was related to the activation of glial cells. In addition, enhanced activation of NRG1 receptor was observed in activated microglia in both ALS patients and the SOD1-mutated ALS mouse model (Song et al., 2012). Coincidentally, another research team also found that NRG1 confers neuroprotection in SOD1-linked ALS mice *via* the restoration of C-boutons (the large cholinergic synapses that innervate spinal α-motor neurons to control their excitability) of spinal motor neurons (Lasiene et al., 2016). In addition, their research also found that the loss of NRG1 expression and C-boutons occurred almost contemporaneously and the expressions of ErbB3 and ErbB4 were reduced in the motor neurons of SOD1-muted ALS mice. Mòdol-Caballero et al. (2020) also found that NRG1 expression was reduced in both ALS patients and the SOD1-mutated ALS mouse model. Overexpression of type III NRG1 can preserve the neuromuscular function of the hindlimbs, improve locomotor performance, increase the number of surviving motoneurons, and reduce glial reactivity in the treated female SOD1 mice. However, it shows no therapeutic efficiency mentioned above in male mice, indicating a possible gender difference. These studies suggest that disruption of the NRG1/ErbB4 pathway is associated with the pathogenesis of ALS and may develop an innovative therapeutic strategy, such as the use of NRGs or their analogs to enhance the function of ErbB4 (Figure 5).

### Neuregulins in Schizophrenia

Schizophrenia is one of the serious psychiatric diseases whose etiology is not fully elucidated. The main clinical characteristics of schizophrenia are perception, thinking, emotion and behavioral disorientations (Owen et al., 2016; Table 4). Although the exact pathogenesis of schizophrenia has not been clarified, it is commonly believed that the pathogenesis and development process of schizophrenia are controlled by genetic and environmental factors. Related studies have found that many common psychiatric diseases have a high genetic tendency and are related to some candidate risk genes, such as schizophrenia (Fusar-Poli et al., 2007), anxiety (Domschke and Dannlowski, 2010), depression (Inkster et al., 2010) and mood disorders (Scharinger et al., 2010). A recent study suggests that some cases of schizophrenia may be associated with abnormal protein accumulation in the brain, similar to AD and other neurodegenerative diseases (Nucifora et al., 2019). Researchers examined postmortem brain samples from 42 schizophrenic subjects and found that half of the brain samples contained significantly higher levels of abnormal proteins, as well as elevated levels of ubiquitin, which is considered as a marker of protein aggregates in neurodegenerative diseases (Ardley and Robinson, 2004; Nucifora et al., 2019).

Studies suggest that NRG1/ErbB4 interactions play a vital role in the pathological mechanism of schizophrenia (Li et al., 2006). With the development of molecular genetics, many genes are related to schizophrenia (Harrison and Weinberger, 2005), of which the most supportive gene is *NRG1* (Harrison and Weinberger, 2005; Norton et al., 2006; Li B. et al., 2007). Thus, the link between the genetic variation of *NRGs* and the increased risk of schizophrenia development may be determined by the wide-ranging effects of NRGs on brain functions (Wang et al., 2009).

The direct and chronic disturbance of NRG1/ErbB4 signal transmission, such as the mutation of *NRG1* or *ErbB4* gene in some schizophrenia patients (Walss-Bass et al., 2006), can lead to the abnormality of glutamatergic synapses and nerve fibers, thus leading to developmental and functional abnormalities. However, the mutation of genes *NRG1* or *ErbB4* may only account for a small part of schizophrenia cases (Harrison and Weinberger, 2005). Other signal transduction or structural components in glutamatergic pathway may be defective, resulting in low function, and may result in the compensatory increase of NRG1/ErbB4 expression and activation levels (Li B. et al., 2007). Dang et al. (2016) investigated the expression of NRG1 and phosphorylated activation status of ErbB4 and ErbB2 in the prefrontal cortex of rats following chronic administration of antipsychotics such as haloperidol, risperidone and clozapine, demonstrating enhanced NRG1/ErbB signaling in the brain. Moreover, studies have found that NRG1 has an important effect on the differentiation, migration and maturation of γ-aminobutyric acid (GABA) interneurons (Flames et al., 2004; Díez et al., 2014). GABA is an important inhibitory neurotransmitter in the forebrain of mammals. It can inhibit intermediate neurons and is essential for the normal function of the CNS (McBain and Fisahn, 2001). GABA dysfunction involves several neurological diseases, including schizophrenia (Coyle, 2004; Woo et al., 2007; Figure 5). In addition, the synaptic hypothesis about the underlying pathophysiological mechanism of schizophrenia suggests that the dysfunction of glutamatergic system can causally affect the onset of schizophrenia (Coyle and Tsai, 2004; Harrison, 2004; Harrison and Weinberger, 2005). In humans, reducing glutamatergic transmission can mimic schizophrenia symptoms (Pietraszek, 2003), whereas, increasing glutamatergic transmission can reduce schizophrenia symptoms (Javitt, 2004). Schizophrenia patients showthe decreased excitatory synaptic function in the hippocampus and cortex (Abbott and Bustillo, 2006; Gan et al., 2014). Another important feature of schizophrenia is neurodevelopmental abnormality. In the early developmental stage, schizophrenia-like symptoms appear in animals with destroyed fiber connection between hippocampus and frontal cortex (Bertolino et al., 1997; Lipska, 2004; Raper et al., 2014). Human studies have shown that early brain maturation, such as premature delivery or perinatal brain injury, may lead to schizophrenia (Pantelis et al., 2005; Rehn and Rees, 2005). These data integratively indicate that the core features of schizophrenia, such as genetic defects, developmental abnormalities and glutamatergic neuron dysfunction, are interrelated (Figure 5).

In order to reveal the role of NRG2 in the modulation of behaviors with relevance to psychiatric disorders, Vullhorst et al. (2015) found a negative feedback loop between N-methyl-D-aspartate receptors (NMDARs) and NRG2/ErbB4 signaling in GABAergic interneurons. Meanwhile, Yan et al. (2018) investigated the role of NRG2 in dopamine balance and glutamatergic transmission using NRG2 knockout mice, and they found NRG2 knockout mice show higher extracellular dopamine levels in the dorsal striatum but lower levels in the medial prefrontal cortex, and NMDAR synaptic currents are augmented at hippocampal glutamatergic synapses. Although the direct evidence of the role of NRG2 in the etiology of schizophrenia remains to be determined, the findings of NRG2 on neuropsychiatric disorders are still inspiring. In addition, NGR3 variants are more associated with the disease manifestations (especially delusions), which appear to be mediated by quantitative changes in the alternative splicing of the gene consistently (Stefansson et al., 2002). Genetic research in schizophrenia shows that risk variants in *NRG3* are associated with cognitive and psychotic symptom severity, which is accompanied by increased expression of prefrontal cortical NRG3 (Meier et al., 2013). While the function and processing of NRG3 have not been studied as well as its NRG1 homolog, their overlaps in similarity and function allow researchers to identify the roles of NRG3 identical to those of NRG1 in schizophrenia (Fiona et al., 2010). Fallin et al. (2005) reported the association of SNP rs1080293 within the gene associated with schizophrenia in a study of 64 candidate genes, including NRG3. In addition, Benzel et al. (2007) investigated the interaction between SNP genotypes across components of the NRG-ErbB signaling network, and they discovered disease-associated SNPs in ErbB4, NRG1, NRG2, NRG3 and ErbB1, as well as significant evidence of interaction between NRG3 and NRG1. Visibly, NRG3 follows NRG1 into the spotlight of schizophrenia research, showing that the influence of genetic variation may exert on the specificity of a phenotype in addition to the risk for this disease.

## Conclusion and Future Perspectives

Although scientists are looking forward to finding cures for neurodegenerative diseases with complex and unclear pathogenesis, no comprehensive and effective treatments and drugs have been developed. The results of a growing number of studies suggest that NRG1 regulates cell maintenance, differentiation, proliferation, migration, and survival or apoptosis in both neuronal and nonneuronal cell types (Mei and Xiong, 2008). Research on NRGs and their corresponding receptors in both invertebrate and vertebrate models, including Drosophila (Hidalgo et al., 2001), zebrafish (Brown et al., 2017), mouse (Kato et al., 2009), rat (Zhao et al., 2015) and even non-human primate rhesus monkey (Zhao, 2013b), have shown their complex involvement in the development of the nervous system and related diseases.

Current evidence has indicated that NRGs play important roles in neurological disorders such as brain trauma (Fricker et al., 2013) and cerebrovascular diseases (Li Y. et al., 2007), and neurodegeneration-related disorders such as AD (Chang et al., 2016), PD (Carlsson et al., 2011), schizophrenia (Mostaid et al., 2016) and ALS (Liu et al., 2018). At present, the clinical translational research on modulation of NRG-ErbB receptor signaling mainly focuses on the targeted treatment of cancers, such as human breast cancer (Geyer et al., 2006; Hurvitz et al., 2013), colorectal cancer (Yonesaka et al., 2011) and gastric cancer (Sato et al., 2013). However, most of these treatments are based on inhibition of NRG-ErbB receptor signaling (Geyer et al., 2006). Good tolerability has been reported for short-termsystemic administration of recombinant human NRG1 (rhNRG1) in a phase II clinical trial (Gao et al., 2010), supporting the potential translation of NRG-ErbB receptor signaling enhancement in the treatment of neurodegenerative diseases. In spite of this, the uncertain CNS penetrant degree may constitute one of the obstacles impeding the application of recombinant rhNRG. An increasing number of studies have inspiringly used virtual screening techniques to obtain small molecule compounds that may potentially treat AD and PD by targeting key pathogenic proteins (Shinde et al., 2015; Gancia et al., 2017; Patel et al., 2018). Thus, future investigation may focus on sound CNS-penetrant small molecule compounds as NRG substitutes to modulate ErbB receptor signaling in the translational treatment of neurodegenerative diseases.

In order to strengthen the understanding of NRGs in neurodegenerative disease prevention and treatment, further relevant mechanisms need to be clarified in the future. More in-depth future research on NRGs is necessary to improve diagnostic solutions for neurodegenerative diseases, thereby reducing the burden on both patients and families.

## Author Contributions

W-jZ: conceptualization. W-jZ, G-yO, and W-wL: writing—original draft preparation. W-jZ and G-yO: writing—review and editing.

## Conflict of Interest

The authors declare that the research was conducted in the absence of any commercial or financial relationships that could be construed as a potential conflict of interest.
